# Anticancer Efficacy of* Cordyceps militaris* Ethanol Extract in a Xenografted Leukemia Model

**DOI:** 10.1155/2017/8474703

**Published:** 2017-07-06

**Authors:** Jae Gwang Park, Young-Jin Son, Tae Ho Lee, Nam Joon Baek, Deok Hyo Yoon, Tae Woong Kim, Adithan Aravinthan, Sungyoul Hong, Jong-Hoon Kim, Gi-Ho Sung, Jae Youl Cho

**Affiliations:** ^1^Department of Genetic Engineering, Sungkyunkwan University, Suwon 16419, Republic of Korea; ^2^Department of Pharmacy, Sunchon National University, Suncheon 57922, Republic of Korea; ^3^Functional Food & Phytomedicine Research Strategic Project Team, Research Planning & Management Department, Dong-A ST, Yongin 17073, Republic of Korea; ^4^Institute for Bio-Medical Convergence, International St. Mary's Hospital and College of Medicine, Catholic Kwandong University, Incheon, Republic of Korea; ^5^Biochemistry, Kangwon National University, Chuncheon 24341, Republic of Korea; ^6^Department of Veterinary Physiology, College of Veterinary Medicine, Biosafety Research Institute, Chonbuk National University, Iksan 54596, Republic of Korea

## Abstract

*Cordyceps militaris* is used widely as a traditional medicine in East Asia. Although a few studies have attempted to elucidate the anticancer activities of* C. militaris*, the precise mechanism of* C. militaris* therapeutic effects is not fully understood. We examined the anticancer activities of* C. militaris* ethanolic extract (Cm-EE) and its cellular and molecular mechanisms. For this purpose, a xenograft mouse model bearing murine T cell lymphoma (RMA) cell-derived cancers was established to investigate in vivo anticancer mechanisms. MTT [3-(4,5-dimethylthiazol-2-yl)-2,5-diphenyltetrazolium bromide] assay, immunoblotting analysis, and flow cytometric assay were employed to check in vitro cytotoxicity, molecular targets, and proapoptotic action of Cm-EE. Interestingly, cancer sizes and mass were reduced in a* C. militaris*-administered group. Levels of the phosphorylated forms of p85 and AKT were clearly decreased in the group administered with Cm-EE. This result indicated that levels of phosphoglycogen synthase kinase 3*β* (p-GSK3*β*) and cleaved caspase-3 were increased with orally administered Cm-EE. In addition, Cm-EE directly inhibited the viability of cultured RMA cells and C6 glioma cells. The number of proapoptotic cells was significantly increased in a Cm-EE treated group compared with a control group. Our results suggested that* C. militaris* might be able to inhibit cancer growth through regulation of p85/AKT-dependent or GSK3*β*-related caspase-3-dependent apoptosis.

## 1. Introduction

*Cordyceps* species are mushrooms that are mainly produced on hosts such as insects.* Cordyceps* species are useful natural products with a variety of biological activities [1, 2].* Cordyceps militaris*, which is a parasite on moth caterpillar larvae, is used broadly in East Asia as a traditional medicine [3]. However, commercializing this mushroom is difficult because it is very rare in nature. Recently, a cultivation process for* C. militaris* was successfully established [4]. This mushroom has received attention as a health supplement to improve longevity, endurance, and human health and as an alternative medicine to ameliorate diseases including stroke, sore throat, tuberculosis, epilepsy, and cancer [5–7]. Many active compounds such as mannitol, ergosterol, polysaccharides, and cordycepin have been identified from* C. militaris*, and studies with these compounds showed diverse pharmacological activities with antiviral, antioxidative, antidiabetic, anti-inflammatory, antiplatelet aggregation, and anticancer effects [8–12]. Although many systemic studies on biological activities of* C. militaris* have been conducted, the pharmacological value of* C. militaris* has not been fully clarified. Therefore, we used a xenograft mouse model bearing murine T cell lymphoma (RMA) cell-derived leukemia to determine the anticancer activity of* C. militaris* ethanolic extract (Cm-EE) and establish its molecular mechanism.

## 2. Materials and Methods

### 2.1. Materials

*Cordyceps militaris* was provided by Mush-Tech (Chuncheon, Korea). RMA and rat C6 glioma cells were from American Type Culture Collection (Manassas, VA) and 3-(4-5-dimethylthiazol-2-yl)-2-5-diphenyltetrazolium bromide (MTT) and hematoxylin and eosin were from Sigma-Aldrich (St. Louis, MO, USA). FITC-Annexin V Apoptosis Detection Kits were from eBioscience (San Diego, CA, USA). RPMI and fetal bovine serum (FBS) were from GIBCO (Grand Island, NY, USA). Total or phosphospecific antibodies against p85, AKT, glycogen synthase kinase 3*β* (GSK3*β*), and caspase-3 were from Cell Signaling (Beverly, MA, USA).

### 2.2. Cell Culture

C6 glioma and RMA cells were cultured in RPMI 1640 supplemented with 10% heat-inactivated FBS and 1% antibiotics (penicillin and streptomycin) at 37°C under 5% CO_2_. For experiments with C6 cells, trypsin/EDTA solution was used to detach cells and 2 × 10^6^ cells/ml were used.

### 2.3. Drug Treatment

Cm-EE powder was suspended in 0.5% sodium carboxymethyl cellulose (Na-CMC) for in vivo experiments. For in vitro experiments, Cm-EE powder was liquefied in 100% DMSO at 100 mg/ml, and the stock solution was diluted with culture medium, as previously reported [13].

### 2.4. Xenograft Mouse Model Experiment

All animal experiments were carried out in accordance with guidelines of the National Research Council's Guide (IACUC, Republic of Korea) for the Care and Use of Laboratory Animals. The experimental protocol was approved by the Animal Experiments Committee of Sungkyunkwan University. Our xenograft animal model was established using C57BL/6 mice (male, five weeks old; Orient, Republic of Korea), as reported previously [14, 15]. Mice were housed individually on a 12-h day/night cycle at 23–27°C with access to food and water. Mice were randomly divided into two groups (*n* = 14/group): (1) a vehicle-control group (*n* = 10) in which animals were orally administered 0.5% Na-CMC and (2) a Cm-EE treatment group (*n* = 10) in which animals were orally administered Cm-EE (20 mg/kg). Mice were injected with RMA cells (1 × 10^6^ cells per animal) subcutaneously in the back next to the right hind leg. After induction of cancer, oral administration of Cm-EE or vehicle was from day 1 to day 21. Cancer sizes for groups were measured with a standard caliper. Cancer volume was calculated as follows: cancer volume (mm^3^) = [cancer length (mm) × cancer width (mm)^2^]/2. On day 25, mice were sacrificed.

### 2.5. Preparation of Total Lysates from Cancer or Cultured Cells and Immunoblotting

C6 glioma cultured cells or cancer tissues were washed three times in cold PBS with 1 mM sodium orthovanadate. Samples were lysed as previously described [16, 17] via suspension in lysis buffer (20 mM Tris-HCl, pH 7.4, 2 mM EDTA, 2 mM ethyleneglycotetraacetic acid, 50 mM *β*-glycerol phosphate, 1 mM sodium orthovanadate, 1 mM dithiothreitol, 1% Triton X-100, 10% glycerol, 10 *μ*g/ml aprotinin, 10 *μ*g/ml pepstatin, 1 mM benzimide, and 2 mM PMSF) for 30 min with rotation at 4°C. Total lysates were clarified by centrifugation at 13,000 ×g for 10 min at 4°C and stored at −20°C. Total lysates were subjected to Western blotting for total p85, AKT, GSK3*β*, and *β*-actin; phosphoforms of p85, AKT, and GSK3*β*; and cleaved caspase-3. Protein levels were visualized as previously reported [13].

### 2.6. HPLC Analysis

For determination of the active components in Cm-EE, high-performance liquid chromatography (HPLC) was conducted as stated previously [18].

### 2.7. Determination of Viability and Apoptosis

Cytotoxicity of Cm-EE on RMA and C6 cells was evaluated by conventional MTT assays, as previously described [19, 20]. Cells were preincubated in 96-well plates at 1 × 10^5^ cells/well for 18 h and treated with indicated concentrations of Cm-EE (0 to 200 *μ*g/ml) for 24 h. Absorbance was measured using a microplate reader at 540 nm. To assess apoptosis, nucleic acid dye 7-amino-actinomycin D-(7-AAD) and Annexin V were used in flow cytometric assays to verify early apoptotic cells.

### 2.8. Evaluation of Tumorigenesis

C6 cells were grown to a monolayer in 60-mm plates for wound healing assays. Wounds were generated as previously reported [21] by scratching the monolayer with a p200 pipette tip. Variation in the scratched monolayer was observed with a microscope (Olympus, Tokyo, Japan). For the invasion assays, the invasive ability of C6 cells was measured as described previously using Matrigel-coated plates [21]. After incubation with Cm-EE for 24 h, cells were fixed in 4% formaldehyde; hematoxylin and eosin was used to stain cells that successfully penetrated the Matrigel layer [22].

### 2.9. Statistical Analysis

**Figure 1:**
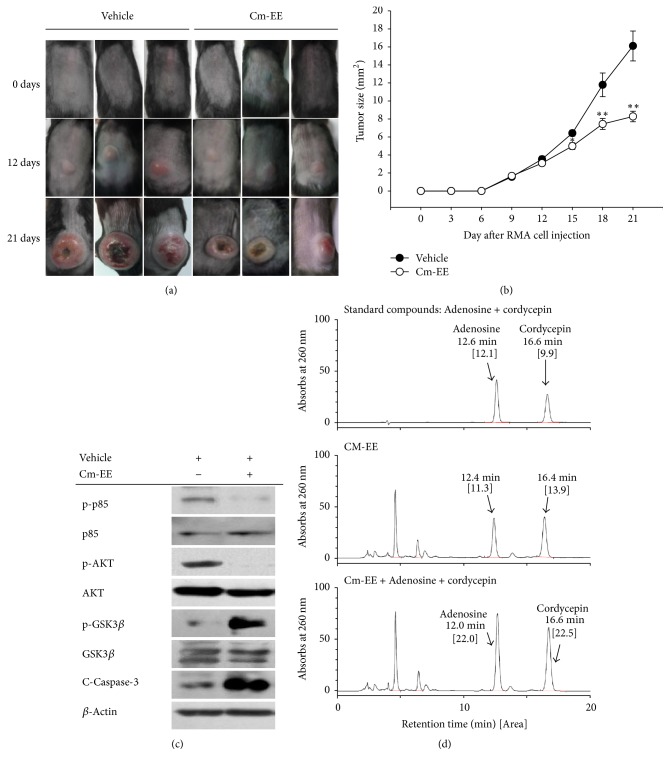
Anticancer activity of Cm-EE in a xenograft mouse model with RMA cell-derived cancer. RMA cells (1 × 10^6^ cells per mouse) were injected subcutaneously into backs next to right hind legs. Mice with RMA cells were sorted into groups (*n* = 10/group) for orally administrated Cm-EE (20 mg/kg) or vehicle. (a) Tumors grown in xenograft mouse model with RMA cell-derived cancer were taken by a digital camera. (b) Induced tumor sizes were measured at indicated days until experiment end. (c) Effect of Cm-EE on proteins in apoptotic pathways was evaluated through determining the levels of total and phosphorylated Akt, p85, GSK3*β*, cleaved caspase-3, and *β*-actin in tumor tissues by immunoblotting analysis. (d) Phytochemical finger printing of Cm-EE was evaluated by HPLC analysis. ^*∗*^*p* < 0.05 and ^*∗∗*^*p* < 0.01 compared with control.

**Figure 2:**
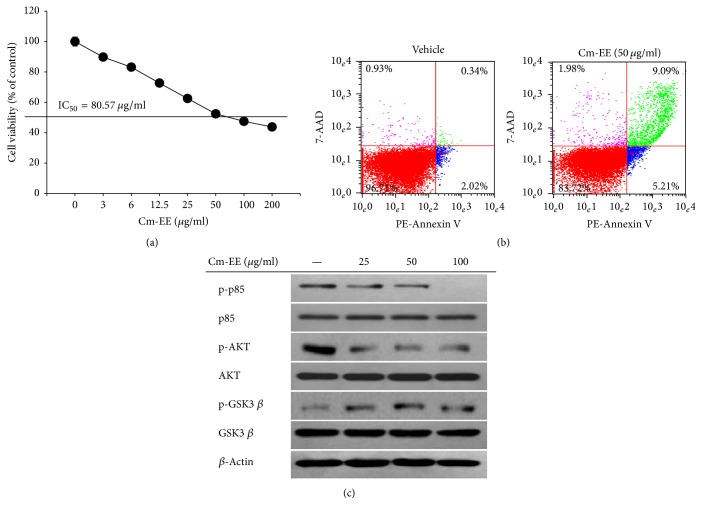
Effect of Cm-EE on apoptotic cell death and survival pathways. (a) RMA cells (2 × 10^6^ cells/ml) were incubated with Cm-EE for 24 h. Viability of RMA cells was then evaluated by MTT assays. (b) Cell apoptosis was determined by Annexin V/7ADD staining. Annexin V^+^ (right lower quadrant) and Annexin V^+^/7AAD^+^ (right upper quadrant) cells indicate early apoptotic and late apoptotic cell levels. The levels of Cm-EE-treated apoptotic cells were evaluated by flow cytometry. (c) Levels of phosphorylated Akt, p85, and GSK3*β* in Cm-EE-treated RMA cells were detected by immunoblotting assays.

All data presented in this paper are mean ± standard deviation (SD) of experiments performed with 10 mice (Figures 1(a), 1(b), and 1(c)) or six replicates (Figure 2). Results were analyzed with Kruskal-Wallis/Mann–Whitney tests for statistical comparisons. *p* < 0.05 was considered statistically significant. All statistical tests were carried out using SPSS (Version 22.0, 2013, IBM Corp., Armonk, NY, USA).

## 3. Results and Discussion

A few studies on the anticancer activity of* C. militaris* have been reported [5–7]. However, the precise reasons for cancer suppression by* C. militaris* have not been elucidated. Therefore, we considered it important to establish the anticancer mechanism of* C. militaris*. We constructed a xenograft mouse model bearing RMA cell-derived cancer to identify the cause of Cm-EE anticancer activity. Over time, tumors in a* C. militaris*-administered group were less developed than tumors in a vehicle-administered group (Figures 1(a) and 1(b)). These results confirmed that cultivated* C. militaris* exhibited anticancer activity similar to other natural products such as Korean red ginseng and wild ginseng [14, 15, 23].

Different* Cordyceps* species have been studied to elucidate pharmacological activity including anti-inflammatory, antioxidant, antifungal, and anticancer activity.* Cordyceps sinensis* species inhibits growth of U937 leukemia, A549 lung cancer, and B16 melanoma cells [24–26].* Cordyceps militaris* inhibits growth of HepG2 liver hepatocellular carcinoma cells [5]. Therefore, previous results indicate that* Cordyceps* species have potent anticancer activity against diverse cancer types including leukemia, melanoma, lung carcinoma, and liver carcinoma.

We investigated the cause of inhibited cancer tissue generation by* C. militaris*. To investigate if Cm-EE anticancer activity was mediated through cell survival and death processes, protein levels were determined by immunoblotting using antibodies against cleaved caspase-3, phospho-p85, phospho-AKT, and phospho-GSK3*β* [27–29]. The levels of phosphorylated p85 and AKT were significantly decreased in a group given Cm-EE. In contrast, levels of phosphorylated GSK3*β* and cleaved caspase-3 were increased in the group administered Cm-EE (Figure 1(c)). The phospho-p85/Akt pathway is reported to be involved in signals related to cell growth and death. Stimulation of this cascade prevents apoptosis [6, 27, 30]. GSK 3*β* is a serine-threonine protein kinase involved in glycogen synthesis. GSK3*β* reportedly has opposing functions, inducing cell apoptosis while maintaining cell survival and proliferation [28]. In our results, accumulation of an active form of GSK3*β* in a group administered Cm-EE seemed to be caused by inhibiting AKT phosphorylation, but the cause was not fully elucidated. We propose that the anticancer activities of Cm-EE were derived from induction of cancer-cell apoptosis through p85/AKT or GSK3*β* signaling pathways. To determine if Cm-EE suppressed cell proliferation directly, we used MTT assays and cultured RMA cells. Cm-EE dose-dependently reduced the viability of RMA cells (Figure 2(a)). To confirm whether cell death was due to apoptosis, flow cytometric assays using Annexin V/7-AAD dyes was conducted. The amount of only Annexin V staining [propidium iodide (PI)/7-AAD negative], indicating early stages of apoptosis [31], was increased with Cm-EE treatment (Figure 2(b)). Levels of proteins related to cell apoptosis, specifically phosphorylated p85, AKT, and GSK3*β*, were similar to results from in vivo experiments (Figure 2(c)).

**Figure 3:**
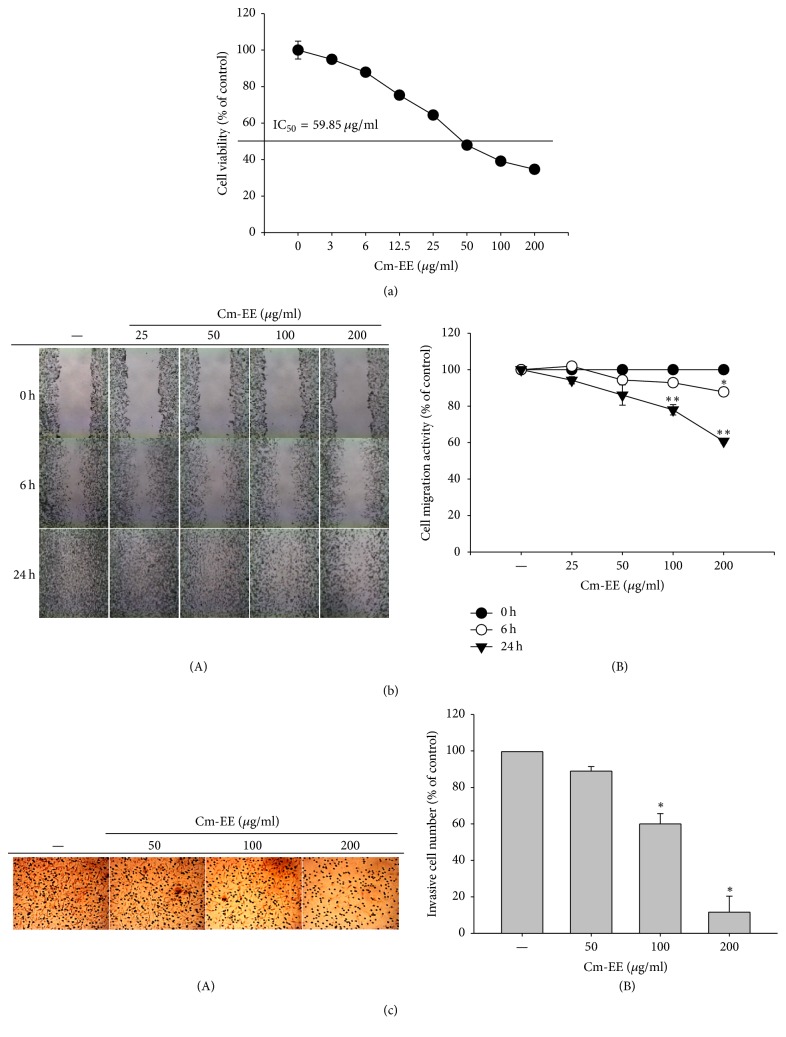
Effect of Cm-EE on tumorigenic responses. (a) C6 glioma cells (2 × 10^6^ cells/ml) incubated with Cm-EE for 24 h. Viability of RMA cells was evaluated by MTT assays. (b) Effect of Cm-EE on migration was measured by wound healing assay. (c) The invasion capacity of C6 cells under Cm-EE exposure was analyzed by hematoxylin and eosin staining and quantitatively evaluated by counting the Matrigel layer-invaded cells (B). Photographs were taken with a digital camera (A). Means of migrated and invasive cells were measured by ImageJ software. ^*∗*^*p* < 0.05 and ^*∗∗*^*p* < 0.01 compared with control.

To determine if the anticancer ability of Cm-EE affected growth or death of other cancer cells, we used cell viability tests and invasion and migration assays with C6 glioma cells under Cm-EE treatment conditions. Similar to the previous results of this study, the viability of C6 cells was dose-dependently reduced by Cm-EE (Figure 3(a)). In addition, Cm-EE abated the migration of C6 glioma cells (Figure 3(b)) and blocked invasion (Figure 3(c)). Based on these results, we suggest that* C. militaris* has anticancer activities such as suppression of cell migration and invasion through stimulation of p85/AKT or GSK*β*-related apoptotic procedures. How Cm-EE regulates p85/AKT or GSK*β*-related apoptosis is not fully understood. Therefore, we will study the regulatory mechanism of p85/AKT or GSK*β*-related caspase-3-dependent apoptosis via* C. militaris* in the future. Many active compounds such as mannitol, ergosterol, polysaccharides, and cordycepin have been identified from* C. militaris* [8–12]. However, anticancer mechanism studies using these compounds seem to be insufficient. Based on these results, future studies will determine a more accurate mechanism for the anticancer activity of* C. militaris*.

**Figure 4:**
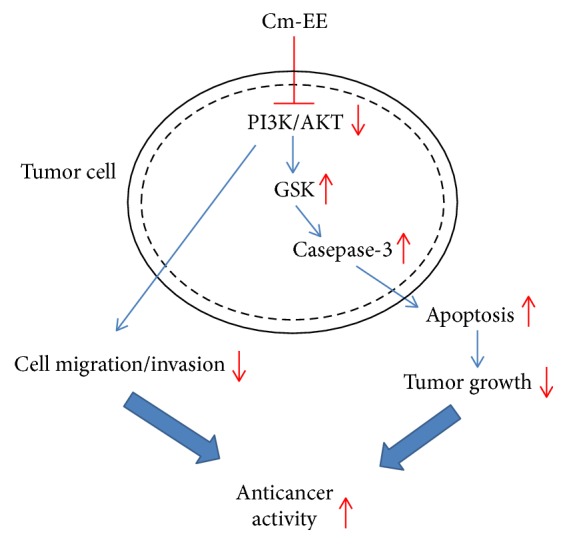
Putative mechanism of Cm-EE-mediated anticancer responses. GSK3*β*: glycogen synthase kinase 3*β*; PI3K: phosphatidylinositol 3 kinase; and Cm-EE:* Cordyceps militaris* ethanolic extract.

In summary, Cm-EE has potent anticancer activity in a xenograft mouse model with RMA cell-derived tumors. Immunoblot analysis showed that apoptotic signaling elements such as p-p85, p-AKT, p-GSK3*β*, and cleaved caspases-3 were regulated as summarized in Figure 4. The number of proapoptotic cells in a* C. militaris*-treated group significantly increased compared with a control group using cultured RMA cells. Cm-EE significantly increased the number of proapoptotic cultured RMA cells and reduced the migration and invasive ability of C6 glioma cells. Our results suggested that* C. militaris* might have a strong ability to inhibit cancer growth through regulation of p85/AKT or GSK3*β*-related caspase-3-dependent apoptosis.
